# Unmet Needs in TAVR: Conduction Disturbances and Management of Coronary Artery Disease

**DOI:** 10.3390/jcm11216256

**Published:** 2022-10-24

**Authors:** Vincent Auffret, Carine Ridard, Nadia Salerno, Sabato Sorrentino

**Affiliations:** 1 CHU Rennes Service de Cardiologie, Université de Rennes 1, Inserm LTSI U1099, 35000 Rennes, France; 2 Vivalto Santé, Centre Hospitalier Privé St-Grégoire, 35760 St-Grégoire, France; 3 Department of Medical and Surgical Sciences, Division of Cardiology, Magna Graecia University, 88100 Catanzaro, Italy

**Keywords:** transcatheter aortic valve replacement, transcatheter aortic valve implantation, coronary artery disease, myocardial infarction, coronary access, coronary revascularization, valve-in-valve, left bundle branch block, high-degree atrioventricular block, permanent pacemaker implantation

## Abstract

Over the past two decades, transcatheter aortic valve replacement (TAVR) swiftly evolved from a disrupting technology towards mainstream therapy in the field of severe symptomatic aortic stenosis. A series of randomized evaluations established its role in treating severe aortic stenosis patients across all surgical risk categories, paving the way for an extension of its indications to younger low-risk patients with a longer life expectancy. Therefore, managing comorbidities and limiting procedural complications, which may affect long-term outcomes, is of paramount importance. Among those, new-onset conduction disturbances and concomitant coronary artery disease remain two of the most debated issues. In the present review, we will discuss the incidence, prognostic impact, and unmet needs of patients with post-TAVR new-onset conduction disturbances and the ongoing challenges posed by the management of concomitant coronary artery disease.

## 1. Introduction

Since its introduction in clinical practice in 2002, transcatheter aortic valve replacement (TAVR) has become the gold standard for the treatment of patients at high surgical risk and is steadily becoming a valuable option for patients deemed at intermediate as well as low operative risk [1]. 

For instance, the longer life expectancy estimated in patients with a lower operative risk, further highlights the importance of limiting procedural complications, such as stroke, myocardial infarction, bleedings, vascular complications, and conduction or rhythmic disturbance, and proposes new challenges for patients’ management [2,3,4,5].

Among those, conduction disturbance requiring permanent pacemaker implantation (PPI) and concomitant coronary artery disease (CAD) remain a matter of debate. Accordingly, this review article will address these topics reporting incidence and prognostic impact as well as the unmet needs of patients with conduction disturbances after TAVR and the challenges in the management of patients with concomitant CAD undergoing TAVR.

## 2. Conduction Disturbances

Conduction disturbances, i.e., high-degree atrioventricular block (HAVB) requiring permanent pacemaker implantation (PPI) and new-onset left bundle branch block (LBBB), represent the most common complication of transcatheter aortic valve replacement (TAVR). Thus, we will briefly review the current knowledge regarding the incidence and clinical impact of these conduction disturbances and reflect upon the challenges posed by these complications going forward in the TAVR setting. An extensive discussion of the mechanisms underlying these conduction disturbances, their natural history, predictors, and management is beyond the scope of the present review and can be found elsewhere [6,7,8,9,10].

## 3. New-Onset Persistent Left Bundle Branch Block

### 3.1. Incidence

With newer-generation devices, rates of 6% to 77% have been reported [6,7]. The incidence of new-onset LBBB with the SAPIEN 3 prosthesis ranges from 6% to 29% [11,12,13,14,15,16,17,18]. The prospective MARE study reported the lowest rate with this iteration of the balloon-expandable device at 6.0% while the randomized PARTNER 3 trial demonstrated a 22% rate of 30 days new-onset LBBB, which was 3-fold higher than the rate of the surgical group [11,19]. Regarding the self-expandable EVOLUT R/PRO system, the MARE study also found a low 8.0% rate of persistent LBBB. Nonetheless, other studies reported an incidence ranging from 18.0% to 44.2% [20,21,22,23]. Regarding other self-expandable systems, the PORTICO valve (Abbott Medical) showed rates of approximately 12% [24,25] while rates of 10.3% to 13.1% have been reported with the ACURATE Neo prosthesis (Boston Scientific) [22,23,26,27].

### 3.2. Clinical Impact

#### 3.2.1. High-Degree Atrioventricular Block and Permanent Pacemaker Implantation

Three meta-analyses reported an approximately 2-fold higher rate of PPI associated with new-onset LBBB at mid-term (≈1 year) follow-up [28,29,30]. A significant impact of new-onset LBBB upon the risk of progression towards HAVB and PPI has consistently been reported either in-hospital [13,31,32,33,34] or at follow-up [32,33,35,36,37,38]. Furthermore, with the exception of the PARTNER I trial analysis [32], the vast majority of studies reported HAVB to be the leading indication (>70%) for PPI at follow-up. Some studies suggested that a QRS duration > 150–160 ms in the setting of new-onset LBBB was associated with a higher risk of late onset HAVB and sudden death [39,40], particularly when associated with a PR interval prolongation (>240 ms) [40,41,42].

#### 3.2.2. Left Ventricular Ejection Fraction (LVEF) and Hospitalization for Heart Failure (HHF)

LBBB may be associated with deleterious ventricular remodeling and deterioration of left ventricular function [43]. Several studies have reported an impaired LVEF recovery after TAVR among new-onset LBBB patients [32,33,35,36,37,38,44,45]. This observation did not translate into a consistently increased risk of hospitalization for heart failure (HHF) in individual studies. Nevertheless, the largest meta-analysis to date reported an increased 1-year HHF risk associated with new-onset LBBB (RR = 1.35; 95% CI: 1.05–1.72) [30].

#### 3.2.3. Mortality

Although it may act through the risk of progression to HAVB (and sudden death) and progressive heart failure as a result of LBBB-induced dyssynchrony, the effect of LBBB on all-cause and cardiovascular mortality has been inconsistent across studies. Regueiro et al. found an increased cardiovascular mortality risk in a meta-analysis of 5 studies, while only a trend was apparent for all-cause mortality combining data from 8 studies [28]. In their updated meta-analysis, Faroux et al. confirmed the deleterious impact upon cardiovascular mortality (RR = 1.46; 95% CI: 1.20–1.72), and unraveled a detrimental impact on all-cause mortality (RR = 1.32; 95% CI: 1.17–1.49) pooling data from 8 studies (5906 patients) and 12 studies (7792 patients), respectively [30].

## 4. Permanent Pacemaker Implantation

### 4.1. Incidence

According to a recent systematic review, post-TAVR rates of PPI with newer-generation devices range from 2.3% to 36.1% [8,46]. Rates were 4% to 24% with the Edwards SAPIEN 3 valve, lower than those reported with the Medtronic EVOLUT R/PRO ranging from 14.7% to 31.3% [21,46]. Interestingly, the risk of PPI at 30 days post-procedure was not significantly different between the TAVR and surgical group in the PARTNER 3 trial, whereas it remained higher after implantation of a self-expandable valve in the EVOLUT Low-Risk trial [19,47]. With the PORTICO valve, rates ranging from 9.8% to 28.1% have been reported [24,48]. Overall, the ACURATE Neo prosthesis demonstrated the lowest rates ranging from 2.3% to 11.5% [26,49]. In the SCOPE I and SCOPE II randomized comparisons, the post-procedural rate of PPI with the ACURATE Neo was similar to the incidence observed with the SAPIEN 3 and significantly lower than the rate reported with the EVOLUT R/PRO, respectively [23,49].

### 4.2. Clinical Impact

Left Ventricular Ejection Fraction and Hospitalization for Heart Failure

The impact of PPI on the evolution of LVEF after TAVR has been inconsistent from one study to another. Some studies suggested a significant decrease in LVEF at follow-up among patients undergoing PPI [36,50,51,52], while others reported no meaningful association [53,54,55,56,57]. These discrepancies may stem from differing pacing indications, pacing dependency, and populations across studies as deleterious effects of right ventricular pacing are more likely to occur in younger patients subjected to a high ventricular pacing percentage over a longer period [45].

PPI post-TAVR has been linked to a higher 1-year risk of HHF in a recent meta-analysis of crude study-level data (RR = 1.18 95% CI: 1.03–1.36) [30]. However, individual studies with a longer follow-up reached conflicting adjusted results [45,54,58].

### 4.3. Mortality

**Figure 1:**
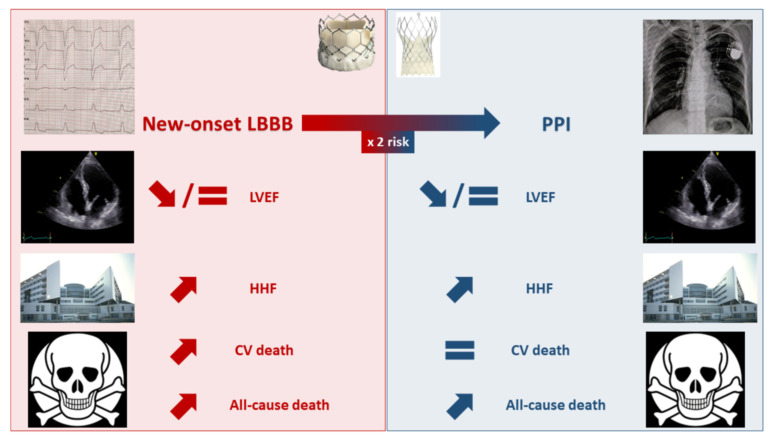
Effects of new-onset left bundle branch block and permanent pacemaker implantation on transcatheter aortic valve replacement outcomes. CV: cardiovascular; HHF: hospitalization for heart failure; LBBB: left bundle branch block; LVEF: left ventricular ejection fraction; PPI: permanent pacemaker implantation.

Faroux et al., reported an increased risk of 1-year all-cause mortality among pacemaker recipients post-TAVR (RR = 1.17 95% CI: 1.11–1.25) [30]. As previously discussed for HHF, long-term studies with a multivariable analysis reached inconsistent results regarding the independent impact of PPI in this finding [45,54,58]. This observation, along with the fact that PPI was not associated with an increased 1-year cardiovascular mortality in the meta-analysis by Faroux et al. [30], raises the issue of potential residual confounding in the association between PPI and post-TAVR mortality. Figure 1 summarizes the effects of new-onset LBBB and PPI on TAVR outcomes.

## 5. Unmet Needs

### 5.1. Pre-Procedural Management

Several studies have demonstrated that a significant proportion of TAVR candidates displayed HAVB or severe bradyarrhythmia during pre-procedural ECG monitoring either with 24-h in-hospital telemetry [59] or with ambulatory systems (patch ECG recorder, mobile telemetry), allowing a prolonged (≥7 days) recording [60,61]. Overall, approximately 3% of patients had HAVB episodes pre-TAVR, among whom 2/3 underwent pre-procedural PPI [62]. Furthermore, almost half of the patients with pre-TAVR bradyarrhythmic events benefited from a treatment change [59,60]. Bradyarrhythmic events were especially frequent among patients with 1st-degree AVB and right bundle branch block (RBBB) occurring in 30% and 47% of them, respectively [60]. Finally, among patients who received a pacemaker post-TAVR, 30% had HAVB episodes pre-TAVR [59,60]. These data suggest that pre-procedural ECG monitoring may be an appealing strategy to streamline patients care, especially those with the highest conduction disturbances risk (e.g., pre-existent RBBB and/or 1st-degree AVB). Nonetheless, further randomized studies are necessary to delineate the optimal indications, duration, clinical impact, and cost-effectiveness of pre-TAVR ambulatory ECG monitoring.

### 5.2. Procedural Management

Several approaches have been proposed in recent years to reduce the occurrence of conduction disturbances during TAVR. Jilaihawi et al. reported the MInimizing Depth According to the membranous Septum (MIDAS) approach, which suggests that a systematic pre-procedural evaluation of the length of the membranous septum below the aortic annulus plane may help tailor the implantation strategy to each patient anatomy [63]. Indeed, the performance of TAVR among increasingly younger and “healthier” patients may imply a need for future coronary re-access. Therefore, the optimal patient-specific implantation depth may result from a compromise between the need to prevent conduction disturbances and to access coronary artery during long-term follow-up. Consequently, in patients with a membranous septum length > 5 mm, considered at low risk of conduction disturbances by Jilaihawi et al., a deeper prosthesis position may be tolerated as long as it does not result in significant paravalvular leak [63]. Moreover, several studies have advocated for a higher implantation of transcatheter heart valves, especially using the cusp-overlap projection, which overlap coronary cusps and isolates the non-coronary cusp, thus providing a better appreciation of the implantation depth [64,65,66]. These reports usually demonstrated an approximately 2-fold lower rate of PPI associated with the use of the cusp-overlap technique. Nonetheless, only one study using a balloon-expandable valve reported a significantly lower rate of new-onset LBBB [64], whereas both studies using self-expandable valves showed a numerically higher rate in the cusp-overlap group compared with the conventional implantation technique [65,66]. Furthermore, the difference in mean implantation depth between the cusp-overlap and conventional implantation groups in these studies was <1 mm questioning the real impact of the cusp-overlap technique in the observed reduction of PPI rates. Finally, a rapid atrial pacing protocol at rates of 70 to 120 beats/min (or until AVB was observed) in 10 beats/min increments for a total of 20 beats at each increment at the end of the procedure was recently proposed to identify patients at low PPI risk within 30 days post-TAVR [67]. This technique demonstrated a 98.7% negative predictive value for 30 days PPI. Nonetheless, it competes with the minimalist approach, usually relying on left ventricular pacing using the stiff guidewire, thus avoiding the central venous puncture and temporary pacemaker placement, which may be associated with some inherent complications [68]. Overall, further studies are needed to delineate the true impact and optimal indications of these procedural techniques of recent emergence.

### 5.3. Post-Procedural Management

One of the main issues regarding post-TAVR conduction disturbances has been the differing management strategies across centers and operators resulting from the lack of consensus, which explain the major differences observed in PPI rates and impact post-TAVR. Several experts’ consensus and guidelines have been published in recent years [9,10,69], which should facilitate a uniform post-procedural management, and allow the performance of large-scale, prospective studies to better describe the long-term impact of these conduction disturbances. Another persistent challenge is the management of conduction disturbances not representing firm PPI indications, i.e., new-onset LBBB and significant PR or QRS prolongation (≥40 ms, especially if PR is >240 ms or QRS > 150 ms). Several studies have demonstrated the safety of using ambulatory ECG monitoring post-TAVR to expedite patients’ discharge and guide PPI in such cases [11,62,70,71,72,73]. Overall, in these studies, delayed HAVB rates have ranged from 5% to 10% and from 10% to 15% approximately, at 30 days and 1-year post-TAVR, respectively. Interestingly, in the largest study to date, encompassing 459 TAVR recipients, the rate of delayed HAVB was higher among patients with new-onset first degree AVB than in patients with new-onset LBBB [72]. Another study demonstrated that the delta between baseline and day 2 post-procedure in PR interval but not in QRS duration was significantly associated with episodes of delayed HAVB [73]. These data suggest that the prolongation of the atrioventricular conduction on the surface ECG may not be a benign occurrence resulting from a supra Hisian injury and that we may need to pay greater attention to this modification. On the other hand, some groups have proposed the use of in-hospital electrophysiological studies (EPS) to guide PPI post-TAVR. Studies focusing on this strategy are usually of limited sample size and used various EPS protocols as well as different HV interval cut-offs to retain an indication for PPI [2,74]. Therefore, the level of evidence seems weaker than for ambulatory ECG monitoring. Nonetheless, these studies have overall demonstrated an excellent negative predictive value of EPS in the post-TAVR setting with a somewhat lower positive predictive value [2,74]. The recent European pacing guidelines granted ambulatory ECG monitoring and EPS-guided strategies (EPS being performed at day 3 post-procedure and an HV interval > 70 ms being used to retain an indication for PPI) the same grade of recommendations in TAVR recipients with new-onset or worsened conduction disturbances [10]. Defining whether ambulatory ECG monitoring or EPS-guided strategies represent the best and more cost-effective option in the post-procedural management of TAVR-related conduction disturbances remains a major unmet need, which is currently addressed by the Clinical Monitoring Strategy Versus Electrophysiology-guided Algorithmic Approach With a New LBBB After TAVI (COME-TAVI) study (NCT03303612). Finally, among TAVR recipients with pre-existent depressed LVEF (<50%) and requiring PPI or with large new-onset LBBB (>150 ms), the role of cardiac resynchronization has not been properly studied yet. Table 1 summarizes ongoing studies regarding conduction disturbances in the setting of TAVR.

## 6. Concomitant Coronary Artery Disease in Patients Undergoing TAVR

### 6.1. Prevalence and Prognostic Impact

The prevalence of CAD in patients undergoing TAVR ranges from ~15% to 80% following the underlying operative risk [75]; a relationship led by the high number of shared risk factors including age, diabetes mellitus, chronic kidney disease, hypercholesterolemia, and hypertension. These patients with exhibit also a high degree of CAD complexity with 50% of multivessel disease [76]. To date, the prognostic impact of CAD on outcomes is controversial. In one meta-analysis pooling 15 studies with more than 8000 patients undergoing TAVR, the presence of CAD (48.7%) was associated with a significant increase in all-cause mortality at 1 year (Odds ratio (OR) = 1.21; 95% CI: 1.07–1.36) [77]. Conversely, a subsequent meta-analysis did not find such an association. However, patients with complex CAD as defined by a SYNTAX score > 22 had greater mortality at 1 year [76]. These contradictory results may be explained by the significant heterogeneity observed across the studies. Furthermore, one bias would be that the patients with severe CAD are usually undergoing surgical aortic valve replacement, unless the estimated surgical risk is high and may require TAVR.

### 6.2. CAD Definition and Hemodynamics Assessment of the Stenosis

Only a limited number of studies provide an objective coronary lesion measurement by quantitative coronary angiography or hemodynamic assessment. In most cases, the indication of revascularization is left at the physician’s discretion, which may have introduced a significant bias. Of interest, since noninvasive ischemia testing is underperformed during TAVR work-out, mainly for patients’ frailty, coronary hemodynamic assessment would be a valuable option to support revascularization-decision making. In this context, both FFR and iFR have been tested in patients with AS [78,79]. In a retrospective single-center study, FFR-guided (*n* = 122/216) revascularization in patients undergoing TAVR showed better outcomes, defined as a composite of cardiac death, myocardial infarction, any coronary revascularization, or disabling stroke compared to the angio-guided group (Hazard ratio (HR) = 0.4; 95% CI, 0.2–1.0). Superiority was even more significant comparing only deferred lesions, based on conventional FFR 0.80 cutoff value (111/142; 78.2%), versus angio-guided percutaneous coronary intervention (PCI) (HR = 0.3; 95% CI, 0.1–0.6) [79]. This last result highlights the possibility to minimize coronary intervention and accordingly antithrombotic strategy in a population with a theoretical high risk of bleeding.

Hemodynamic changes in coronary blood flow and other coronary physiological parameters after TAVR were assessed by Vendrik J. and colleagues in 13 patients with AS [80]. Interestingly, hyperemic coronary flow velocity increases acutely after TAVR and continues to rise to 6-month follow-up. Conversely, resting diastolic flow, and consequently, iFR is not affected by severe AS and remains unchanged pre-TAVR, post-TAVR, and at 6-month follow-up. Yamanaka et al. [81] showed a good correlation between FFR and iFR in discriminating myocardial ischemia with perfusion scintigraphy, identifying iFR < 0.82 as the new cut-off for an FFR < 0.75 and myocardial ischemia on perfusion scintigraphy. This cut-off of 0.82 for iFR was further confirmed in another study [82]. The ongoing trials FAITAVI (Functional Assessment in TAVI), NOTION-3 (Revascularization in Patients Undergoing Transcatheter Aortic Valve Implantation), and TAVI-PET (Correlation of FFR and iFR With Cardiac PET Perfusion in Patients with Severe Aortic Valve Stenosis) will provide information to comprehend the role of FFR/iFR in this group of patients.

### 6.3. Heterogeneity in Endpoint Definition

The heterogeneity in endpoint definition and lack of long-term follow-up (>3 y.o.) may fail to discriminate CAD as an independent prognostic determinant rather than a simple marker of comorbidity and increased risk status. Looking into perspective, a longer follow-up becomes even more important when younger and lower-risk patients are treated with TAVR.

In this perspective, Minten and colleagues [83] recently published the results of a large prospective single-center observational study, evaluating the interplay between CAD complexity, its management, and long-term outcomes after TAVR. This study, including 604 all-comers patients from 2008 to 2020, has shown that: (1) 346 patients presenting CAD had significantly worse all-cause death (55.1% vs. 67.9%; HR = 1.41; *p* = 0.022) and cardiovascular death (74.9% vs. 84.9%; HR = 1.62; *p* = 0.039) as compared with those without CAD at 5-year follow-up; a difference that was not significant at shorter term (1–3 years) of follow up; (2) the presence of complex CAD, defined as syntax score >22, was an independent predictor for cardiovascular death at 5 years after TAVR; and (3) neither pre-TAVR PCI nor completeness of revascularization seemed to reduce the increased risk for these adverse clinical outcomes. Despite the small number of patients retained after 2 years of follow-up (~40%), this study highlights the importance of a prolonged observational period to bring out the prognostic impact of CAD and its complexity and confirmed the uncertainty about the timing and completeness of revascularization.

The ACTIVATION (PercutAneous Coronary inTervention prIor to transcatheter aortic VAlve implantaTION) trial evaluated the impact of revascularization in 235 patients with significant CAD, assigned to receive PCI or no PCI before TAVR. At 1 year, rates of all-cause mortality or rehospitalization were similar between the groups, occurring in 41.5% of patients who underwent PCI and 44% of those who did not. Unfortunately, the noninferiority margin was not met (difference: −2.5%; 1-sided upper 95% confidence limit: 8.5%; 1-sided noninferiority test *p* = 0.067). However, in the as-treated analysis, the difference was −3.7% (1-sided upper 95% confidence limit: 7.5%; *p* = 0.050), with no difference in the rates of stroke, myocardial infarction, or acute kidney injury, with higher rates of any bleed in the PCI arm [84].

Similar results were observed in a recently published meta-analysis pooling 24 studies and 12,182 TAVR patients of which 4110 (33.7%) underwent pre-TAVR PCI, with 30-day (OR = 1.19; 95% CI: 0.91–1.55) as well as 1-year mortality (OR = 1.12; 95% CI: 0.95–1.31) being comparable. Finally, as observed in the ACTIVATION trial, this meta-analysis also found an increased risk of life-threatening bleeding at 30 days [85].

### 6.4. Optimal Timing of Revascularization

Evidence supporting PCI before or after TAVR is scarce, and the optimal timing of PCI in patients scheduled for TAVR is still a matter of debate. The 2020 ACC/AHA Guideline for the management of patients with valvular heart disease recommends PCI before TAVR for the treatment of left main or proximal CAD [86]. Conversely, the 2021 guidelines from the European society of cardiology recommend revascularization based on clinical presentation, coronary anatomy, and extent of myocardial at risk [87] (Table 2).

**Figure 2:**
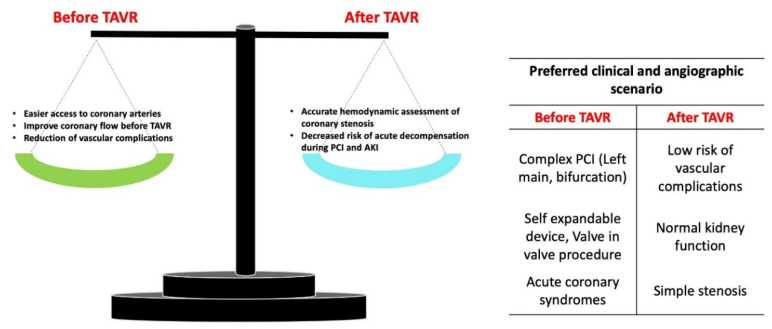
Advantages and disadvantages of performing PCI before or after transcatheter aortic valve replacement. PCI: percutaneous coronary intervention; AKI: acute kidney injury.

Figure 2 illustrates advantages and disadvantages of performing PCI before or after TAVR.

A retrospective registry including 55,754 patients treated with TAVR from 1 January 2010 to 30 June 2019, provide one of the largest available landscapes on the current clinical practice in this subset of patients. In this population based on the national hospitalization PMSI (Programme de Médicalisation des Systèmes d’Information) database covering hospital care from the entire French population, a total of 8613 (15%) subjects had a PCI from 90 days before to 90 days after the TAVR procedure. In most cases, PCI was performed before TAVR (*n* = 8384) and was more frequently performed in the post-TAVR subgroup only for acute MI. Of interest is that after propensity score matching, similar outcomes were observed between PCI first versus post-TAVR at 30 days as well as 1 year of follow-up (459 ± 569 days) [88]. This registry confirms that most of the patients with CAD are treated before TAVR, despite this being associated with an increased risk of acute kidney injury, bleeding, and vascular complications [89,90]. This is probably because the selective ostia re-engagement remains a matter of concern for interventional cardiologists, who prefer to perform PCI after TAVR only in limited cases such as ACS.

Challenges in ostium re-engagement may be related to anatomical, procedural, and prosthesis features. Sinotubular junction dimension, sinus height, leaflet length and bulkiness, sinus of Valsalva width, and coronary height are anatomical key features that may or may not facilitate ostia engagement. On the other hand, commissural tab orientation, sealing skirt height, and valve implantation depth represent the procedural features that may influence engagement [91]. The single-center prospective RE-ACCESS study has shown that Evolut Valve (Medtronic, Minneapolis, MN, USA), and prosthesis implantation depth were predictors of unsuccessful coronary cannulation, while the ALIGN TAVR study [92] showed that orienting the Evolut delivery catheter with the flush port positioned at 3 o’clock and tracking the Evolut hat marker at the outer curve of the thoracic aorta reduced the incidence of severe coronary artery overlap from 38% to 24%.

On a valve type point of view, Evolut prosthesis may not allow an easy engagement compared with Sapien valves (Edwards Lifesciences, Irvine, CA, USA) [91,93,94]. Indeed, the self-expandable design extends beyond coronary ostia with a high risk to hinder selective coronary cannulation by the neo-commissure of the prosthesis. Conversely, the lower length and the wider upper row of the Sapien valve stent frame compared to the Evolut simplify selective coronary cannulation [91,93].

In summarizing, according to the current evidence, CAD management in patients undergoing TAVR should consider clinical characteristics, anatomical valve structure, type of valve chosen, and finally the complexity of CAD.

Finally, several ongoing studies will provide further information about the optimal management of CAD in patients undergoing TAVR. In particular, the NOTION-3 (NCT03058627) and the FAITAVI (NCT03360591) randomized trials will evaluate the role of FFR-guided complete revascularization on outcomes. The COMPLETE TAVR (NCT04634240) trial will randomize 4000 patients with significant CAD after successful TAVR to PCI versus medical therapy alone.

## 7. Conclusions

Despite some discrepancies in the available literature, a detrimental impact of conduction disturbances, i.e., new-onset LBBB and PPI, on mid-to-long-term outcomes post-TAVR is likely. Alongside conduction disturbances, coronary artery disease is another element of concern for patients undergoing TAVR. Risk stratification, stenosis evaluation, and timing of intervention are key points to face during TAVR work-out. However, several issues remained unaddressed throughout the TAVR workflow and should be the focus of future prospective studies to reduce the burden of post-TAVR conduction disturbances and provide even more evidence for the treatment of coexisting CAD.

## Figures and Tables

**Table 1:** Ongoing studies regarding conduction disturbances in the setting of transcatheter aortic valve replacement.

NCT Number	Study Name	Planned Number of Patients	Target Population	Design and Timing	Intervention	Main Outcomes
NCT03810820	Remote ECG Monitoring of TAVI Patients	240	Consecutive candidates to outpatient TAVR	Observational, prospective, pre and post-procedure	Mobile cardiac telemetry (m-CARDS) before and after TAVR	Feasibility/patients’ adherence. Timeliness of medical assessment. Any new conduction disturbances up to 30 days.
NCT04139616	PROMOTE	2000	All TAVR recipients without prior pacemaker	Observational, prospective, post-procedure	Application of a pre-specified algorithm for the management of conduction disturbances post-TAVR	Implementation of the algorithm. Incidence of PPI and sudden cardiac death up to 1 year
NCT02659137	HESITATE	100	All TAVR recipients without pre-existent conduction disturbances	Observational, prospective, per and post-procedure	EPS during the procedure	Measurement of the HV interval upon occurrence of a LBBB. Location of the LBBB
NCT04454177	SMART TAVR	100	All TAVR patients	Observational, prospective, post-procedure	Huawei smart watch	Composite of death and rehospitalization, rates of conduction disturbances and PPI at 30 days
NCT04489095	Conduction Disease After Transcatheter Aortic Valve Replacement	200	All TAVR recipients without prior pacemaker	Prospective, observational, per and post-procedure	EPS immediately before and after TAVR and the next day	Correlation between delta values of EPS findings and high-grade conduction disturbances at 1 year
NCT02482844	LBBB-TAVI	200	TAVR recipients with new-onset LBBB	Observational, prospective, post-procedure	EPS with PPI if HV interval >70 ms and implantable cardiac monitoring if <70 ms.	Incidence of HAVB at 1 year
NCT04128384	HOM TAVI	200	All TAVR recipients without prior pacemaker	Observational, prospective, per and post-procedure	Limited EPS including HV- and AH-intervals measurements pre- and post-TAVR	Incidence of HAVB and persistence of new-onset LBBB at 2 years
NCT03303612	COME TAVI	200	TAVR recipients with new-onset LBBB	Randomized, prospective, post-procedure	Group 1: EPS-based strategy Group 2: Clinical follow-up with implantable cardiac monitoring.	Incidence of the composite of cardiovascular hospitalization, syncope or death at 1 year. Incidence of HAVB at 1 year. Cost-effectiveness.
NCT02768064	PAMIT	120	All TAVR recipients without prior pacemaker	Randomized, prospective, per and post-procedure	Experimental: Flexible screwed temporary pacemaker Active Comparator: Stiff standard temporary pacemaker	Incidence of pericardial effusion, electrode dislocation, and other temporary pacing complications at 1 week
NCT04482816	PHYS-TAVI	24	TAVR recipients with HAVB pacing indication after TAVR and LVEF > 50%	Randomized, prospective, post-procedure	Experimental: Physiological (His system) pacing Active Comparator: Right ventricular pacing	Composite of survival, NYHA improvement and >25% increase in the 6MWT at 1 year. LVEF at 1 year.

6MWT: 6 min walking test; EPS: electrophysiological study; HAVB: high-degree atrioventricular block; LBBB: left bundle branch block; LVEF: left ventricular ejection fraction; NYHA: New York heart association; PPI: permanent pacemaker implantation.

**Table 2:** Management of CAD in patients undergoing TAVR.

	ESC Guidelines	AHA Guidelines
Diagnosis	Coronary angiography is recommended before TAVR; coronary CTA may be considered in patients with low risk for CAD, or in patients in whom conventional ICA is technically not feasible or associated with increased risk.	Contrast-enhanced CTA (in patients with a low pretest probability for CAD) or an invasive coronary angiogram is recommended (Class 1)
Treatment	PCI should be considered in patients undergoing TAVR and coronary artery diameter stenosis > 70% in proximal segments (Class IIa, Level C).	Revascularization by PCI before TAVR is reasonable in patients with significant left main or proximal CAD with or without angina (Class 2a).
	Patients with severe symptomatic aortic stenosis and diffuse CAD unsuitable for revascularization should receive optimal medical therapy and undergo SAVR or TAVR according to individual characteristics.	In patients with significant CAD (luminal reduction > 70% diameter, FFR < 0.8, iFR < 0.89) consisting of complex bifurcation left main and/or multivessel CAD with a SYNTAX score > 33, SAVR and CABG are reasonable and preferred over TAVR and PCI (Class 2a).
	Percutaneous coronary intervention (PCI) and TAVR may be undertaken as combined or staged procedures according to the clinical situation, pattern of CAD, and extent of myocardium at risk	

AHA: American Heart Association; CAD: coronary artery disease; CTA: computed tomography angiography; ESC: European Society of Cardiology; ICA: invasive coronary angiography; PCI: percutaneous coronary intervention; SAVR: surgical aortic valve replacement; TAVR: transcatheter aortic valve replacement.
